# Supplementary material for: Multilocus sequence typing and antibiotic resistance of Aeromonas isolated from freshwater fish in Hebei Province
Source: PLoS One. 2024 Mar 27;19(3):e0298745. doi: 10.1371/journal.pone.0298745 (PMC10971578; doi:10.1371/journal.pone.0298745)
Supplement: Attachment — Submitted filename: Author to respond reviewer 1 - PLOS ONE.docx [file pone.0298745.s006.docx]

**Response to Reviewer 1 Comments**

-The current study is interesting; however, the authors should address the following comments to improve the quality of the manuscript:

-The manuscript should be revised for English editing and grammar mistakes.

- Please write the scientific names of bacterial pathogens and genes in the correct form all over the manuscript and the references section.

**Title:**

**Point 1.** I think the work would benefit from the title that contains the main conclusion of the study (should be derived from the conclusion). Please modify the title.

**Response 1:** We thank the Reviewer for pointing this out. We have revised the title corresponding to line 1-2 of page 1 as follows:

(Page 1, Line 1-2): “Multilocus Sequence Typing and Antibiotic Resistance of *Aeromonas* Isolated from Freshwater Fish in Hebei Province”

**Abstract:**

**Point 2.** The abstract must illustrate the used methods and the most prevalent results (give more hints about methods and results). Besides, rephrase the aim of the work and the main conclusion of your findings.

**Response 2:** We have revised the Abstract corresponding to line 32-57 of page 2 as follows:

(Page 2, Line 32-57): “*Aeromonas* spp. are the opportunistic pathogens that infect both aquatic and terrestrial homeotherms. They were commonly present in aquatic environments, including effluent, tap water, marine, river, and lake, where they are often isolated from aquatic animals, including fish, molluscs, and crustaceans. The *Aeromonas* infections can cause sepsis, ulcer, and other symptoms, resulting in the death of massive aquatic animals. Therefore, the prevention and control of *Aeromonas* is of great significance for the healthy development of aquaculture. In this study, we used modern molecular methods to enhance disease control of *Aeromonas* isolates from freshwater fish in Hebei Province. A total of 130 *Aeromonas* spp. isolates were isolated from freshwater fish farms in Hengshui, Handan, and Shijiazhuang and all 130 *Aeromonas* spp. isolates were sequenced for species identification. Of the 130 *Aeromonas* spp. isolates, 104 isolates were successfully sequenced, and BLAST analysis showed that *Aeromonas veronii* was predominant in freshwater fish farms in Hebei Province. In addition, 26 antibiotic resistance profiles were obtained from 102 fully cultured isolates among the 104 *Aeromonas* spp. isolates whose species was primarily identified, and 44 multidrug-resistant bacteria among the 102 isolates were identified using an antibiotic susceptibility test. Using the Multilocus Sequence Typing (MLST) method, 33 out of 44 multidrug-resistant isolates with 14 non-*Aeromonas* reference strains were selected for phylogenetic and MLST analysis, and all 33 multidrug-resistant isolates were *A. veronii*. A total of 30 new Sequence Types (STs) were obtained by comparing concatenated sequences (*gyrB*-*groL*-*gltA*-*metG*-*ppsA*-*recA*) on PubMLST website. Furthermore, recombination event analysis detected using RDP5 and ClonalFrameML software 42 and 49 recombination events, respectively, and 22 recombination events were validated by four or more algorithms. Since mutation and recombination events increase clonal diversity and single housekeeping gene sequence alignments are limited for identifying species, we propose the use of multiple concatenated sequence loci to increase discriminatory power. In addition, we propose that the MLST method is an appropriate technique to study and develop the resistance mechanisms of multidrug-resistant *Aeromonas* and to identify *Aeromonas* systematically in complex samples obtained from the environment.”

**Point 3.** Add the full expression before the abbreviations.

**Response 3:** We have corrected as Reviewer’s suggestion.

**Introduction: (it needs to be more informative):**

**Point 4.** Give a hint about the virulence factors and the mechanism of disease occurrence, and infecions caused by Aeromonas spp.

**Response 4:** We have revised the Introduction corresponding to line 64-75 of page 3 as follows:

(Page 3, Line 64-75): “*Aeromonas* spp. possess various virulence factors such as degradative enzymes, surface polysaccharides (capsule, lipopolysaccharide, and glucan), S-layers, iron-binding systems, exotoxins, secretion systems, fimbriae, other nonfilamentous adhesins, and flagella [A17, A18]. Various virulence factors of Aeromonas spp. cause immunocompromised organisms through resistance to host defense mechanisms, leading to diseases such as septicemia, ulcers, and visceral erosion and directly damaging host cells, resulting in Aeromonas infection and cell death [3, A19]. In addition, antibacterial and antioxidant molecules in the fish body provide self-defense against invading Aeromonas spp and virulence factors, but the end product of this is reactive oxygen species (ROS), which can cause host cell damage and stress [A20]. Aquatic animals such as fish, shrimp, and amphibians are particularly susceptible to Aeromonas infection due to Aeromonas spp being the most widespread species in the aquatic environment, which can result in high mortality [4, 5].”

**Point 5.** The authors should illustrate the public health importance concerning the emergence of multidrug-resistant (MDR) bacterial pathogens that reflect the necessity of new potent and safe antimicrobial agents. Several studies proved the widespread MDR- bacterial pathogens;

Authors could add the following paragraph:

Multidrug resistance has been increased all over the world that is considered a public health threat. Several recent investigations reported the emergence of multidrug-resistant bacterial pathogens from different origins that increase the necessity of the proper use of antibiotics. Besides, the routine application of the antimicrobial susceptibility testing to detect the antibiotic of choice as well as the screening of the emerging MDR strains. You are advised to cite the following valuable studies:

1. PMID: 36819057

2.PMID: 36365013

3. PMID: 32235800

4. PMID: 36439817

5. PMID: 35971557

6. PMID: 35532863

7. PMID: 31170450

**Response 5:** We have revised the Introduction corresponding to line 80-84 of page 3 as follows:

(Page 3, Line 80-84): “Multiple drug resistance (MDR) has been increased all over the world that is considered a public health threat [15-17]. Several recent investigations reported the emergence of multidrug-resistant bacterial pathogens from different origins that increase the necessity of the proper use of antibiotics [18, 19]. Besides, the routine application of the antimicrobial susceptibility testing to detect the antibiotic of choice as well as the screening of the emerging MDR strains [20, 21].”

**Point 6.** Illustrate the mechanism of action different virulence factors of Aeromonads.

**Response 6:** As in ‘Point 4’, We have corrected as Reviewer’s suggestion.

**Point 7.** Rephrase the aim of the work to be clear and better sound.

**Response 7:** We have revised the Introduction corresponding to line 124-127 of page 5 as follows:

(Page 5, Line 124-127): “Therefore, this study aims to investigate the antibiotic resistance of *Aeromonas* from aquatic organisms in Hebei Province, China, and verify whether MLST technology is suitable for classifying multidrug-resistant *Aeromonas* isolated from the environment.”

**Material and methods:**

**Point 8.** Support all methods with updated specific references.

**Response 8:** We sincerely appreciate the valuable comments. We have checked the literature carefully and added more references as follows:

(Page 4, Line 120, Page 8, Line 205 and Page 14, Line 372): The Clinical and Laboratory Standards Institute Subcommittee on Antimicrobial Susceptibility Testing: Background, Organization, Functions, and Processes

(Page 6, Line 148): boiling method: Ip M , Liyanapathirana V , Ang I ,et al. Direct Detection and Prediction of All Pneumococcal Serogroups by Target Enrichment-Based Next-Generation Sequencing[J]. Journal of Clinical Microbiology, 2014, 52(12):4244-52.

(Page 7, Line 176): BLAST: Altschul SF, Gish W, Miller W, Myers EW, Lipman DJ. Basic local alignment search tool. J Mol Biol. 1990 Oct 5;215(3):403-10.

(Page 7, Line 185): The Kimura 2-parameter (K2P) model: Kimura M. A simple method for estimating evolutionary rates of base substitutions through comparative studies of nucleotide sequences. J Mol Evol. 1980 Dec;16(2):111-20.

(Page 8, Line 196): Kirby-Bauer disk diffusion susceptibility test: Kaspar H M D , Koss M J , He L ,et al.Antibiotic susceptibility of preoperative normal conjunctival bacteria[J]. American Journal of Ophthalmology, 2005, 139(4):730-733.

**Point 9.** Add the company, city, and country of the used chemicals and reagents.

**Response 9:** We sincerely thank the Reviewer for careful reading. As suggested by the reviewer, we have added:

(Page 6, Line 143- 144): “RS medium (Hopebio, Qingdao, China)”

(Page 6, Line 149): “Brain heart infusion broth (TOPBIO, Yantai, China)”

(Page 7, Line 170 and Page 9, Line 242-243): “gel imaging system (Bio-Rad, CA, USA)”

(Page 8, Line 196-197): “The above reagents are purchased from Hangzhou Microbial Regent Co., Ltd. (Hangwei, Hangzhou, China)”

(Page 8, Line 202): “Muller Hinton Agar (Hopebio, Qingdao, China)”

**Point 10.** Bacterial Strains: Please illustrate the methods used for isolation and identification of Aeromonas spp.: Besides, specific references should be added.

**Response 10:** We have revised the Materials and Methods corresponding to line 143-146 of page 6 as follows:

(Page 6, Line 143-146): “The samples were aseptically cultured on Aeromonas isolation medium RS medium using the streak plate method at 30°C for 18-24 hours, after that, the sterile inoculation loops were used to pick up the single yellow colonies and streak them on clean RS medium to obtain pure cultures.”

We have added more references:

(Page 7, Line 165): primer 16S rRNA : Frank J A, Reich C I, Sharma S, et al. Critical Evaluation of Two Primers Commonly Used for Amplification of Bacterial 16S rRNA Genes [J]. Appl Environ Microbiol, 2008, 74(8): 2461-2470.

(Page 7, Line 165): primer gyrB: Martino M E, Fasolato L, Montemurro F, et al. Determination of microbial diversity of Aeromonas strains on the basis of multilocus sequence typing, phenotype, and presence of putative virulence genes [J]. Appl Environ Microbiol, 2011, 77(14): 4986-5000.

**Point 11.** Add the company, city, and country of the used bacterial media and reagents that were used in the biochemical identification of isolates. Also, enumerate all used biochemical reactions.

**Response 11:** As in ‘Point 9’, We have corrected as Reviewer’s suggestion.

**Point 12.** Antimicrobial susceptibility testing: Please, explain in detail

Add the names of the antimicrobial classes.

**Response 12:** We have revised the Materials and Methods as follows:

(Page 8, Line 189-197): “A total of 13 antibiotics from 10 classes were selected for Kirby-Bauer disk diffusion susceptibility test: Penicillin G: amoxicillin (AMX, 30 ug); Chloramphenicol: florfenicol (FLR, 30 ug); Cephalosporin: cefradine (CED, 30 ug), cefuroxime (CXM, 30 ug), ceftazidime (CAZ, 30 ug), cefepime (FEP, 30 ug); Monocyclic *β*-lactams: aztreonam (ATM, 30 ug); Carbapenem: meropenem (MEM, 10 ug); Quinolone: ciprofloxacin (CIP, 5 ug); Sulfonamide: co-trimoxazole (SXT, 1.25/23.75 ug (Sulfamethoxazole (SMZ, 1.25 ug) / Trimethoprim (TMP, 23.75 ug)); Aminoglycoside: amikacin (AMK, 30 ug); Polypeptide: polymyxin B (POL, 300units); Tetracycline: tetracycline (TCY, 30 ug) [44, 45]. The above reagents are purchased from Hangzhou Microbial Regent Co., Ltd. (Hangwei, Hangzhou, China).”

(Page 8, Line 199-203): “The 102 *Aeromonas* isolates were inoculated on BHI (TOPBIO, Yantai, China) agar overnight and single-colony was picked and resuspended in 0.9% saline. The concentration of *Aeromonas* was adjusted to 1.5 x 10^8^ CFU/mL according to the McFarland scale, evenly coated on Muller Hinton Agar (Hopebio, Qingdao, China) and incubated at 28°C for 18-24 hours in an inverted position.”

**Point 13.** The authors are advised to classify the tested isolates to MDR , XDR, and PDR as described by Magiorakos et al. Also, the MAR index was determined according to Krumperma.

Magiorakos AP, Srinivasan A, Carey RB, Carmeli Y, Falagas ME, Giske CG, et al. Multidrug-resistant, extensively drug-resistant and pandrug-resistant bacteria: An international expert proposal for interim standard definitions for acquired resistance. Clin Microbiol Infect. 2012; 18:268–81. doi:10.1111/j.1469-0691.2011.03570.x.

Krumperman PH. Multiple antibiotic resistance indexing of Escherichia coli to identify high-risk sources of fecal contamination of foods. Applied and Environmental Microbiology. 1983;46(1):165-70.

**Response 13:** We have corrected as Reviewer’s suggestion. We have created a new “Table 4”

We have added more references:

(Page 11, Line 298): Magiorakos AP, Srinivasan A, Carey RB, Carmeli Y, Falagas ME, Giske CG, et al. Multidrug-resistant, extensively drug-resistant and pandrug-resistant bacteria: An international expert proposal for interim standard definitions for acquired resistance. Clin Microbiol Infect. 2012; 18:268–81. doi:10.1111/j.1469-0691.2011.03570.x.

(Page 11. Line 300): Krumperman PH. Multiple antibiotic resistance indexing of Escherichia coli to identify high-risk sources of fecal contamination of foods. Applied and Environmental Microbiology. 1983;46(1):165-70.

Table 4. Drug resistance spectrum of 102 Aeromonas isolates.

| **Strains** | **Resistance Spectrum** | **Type** | **MDR** | **MAR** | **Amount** | **Percentage** |
| --- | --- | --- | --- | --- | --- | --- |
| WX8, WX13, WX10, 216182, 21613, 21609, 21603, 21602, 21514, 21513, 21512, 21411, 21404, 21403, 20812, 20808, 20418, 20411 | AMX | A | 0 | 0.08 | 18/102 | 17.65 |
| WX7, WX5, WX3, WX1, 216062, 216061, 201008, 21618, 21615, 21611, 21608, 21605, 21508, 21505, 21504, 21503, 21407, 21405, 21401, 20904, 20903, 20902, 20815, 20814, 20813, 20803, 20709, 20616, 20611, 20506, 20505, 20414, 201009, | CED/AMX | B | 0 | 0.15 | 33/102 | 32.35 |
| 20710 | TCY/AMX | C | 0 | 0.15 | 1/102 | 0.98 |
| 21501, 20417 | SXT/AMX | D | 0 | 0.15 | 2/102 | 1.96 |
| 20507 | CIP/AMX | E | 0 | 0.15 | 1/102 | 0.98 |
| 21506, 20907, 20906 | MEM/AMX | F | 0 | 0.15 | 3/102 | 2.94 |
| 21601, 21511, 20905, 20804, 20712 | TCY/CED/AMX | G | 5 | 0.23 | 5/102 | 4.90 |
| WX14, 20416 | POL/CED/AMX | H | 2 | 0.23 | 2/102 | 1.96 |
| 21616, 21515, 20809, 20415 | SXT/CED/AMX | I | 4 | 0.23 | 4/102 | 3.92 |
| 201001 | SXT/TCY/AMX | J | 1 | 0.23 | 1/102 | 0.98 |
| 201010, 21621, 21619, 21510, 21509, 21507, 21409, 20910, 20801, 20705, 20617 | MEM/CED/AMX | K | 11 | 0.23 | 11/102 | 10.78 |
| 20703 | FLR/CED/AMX | L | 1 | 0.23 | 1/102 | 0.98 |
| WX6, 201007, 201004, 20901, 20503 | MEM/TCY/CED/AMX | M | 5 | 0.31 | 5/102 | 4.9 |
| 201003 | MEM/SXT/TCY/AMX | N | 1 | 0.31 | 1/102 | 0.98 |
| 21607 | MEM/SXT/AMK/AMX | O | 1 | 0.31 | 1/102 | 0.98 |
| 201002 | FLR/TCY/CED/AMX | P | 1 | 0.31 | 1/102 | 0.98 |
| 21410 | FLR/SXT/CED/AMX | Q | 1 | 0.31 | 1/102 | 0.98 |
| 21408 | FLR/MEM/CED/AMX | R | 1 | 0.31 | 1/102 | 0.98 |
| 21614 | SXT/AMK/CXM/CED/AMX | S | 1 | 0.39 | 1/102 | 0.98 |
| 21412 | MEM/SXT/POL/CED/AMX | T | 1 | 0.39 | 1/102 | 0.98 |
| 20471 | FLR/MEM/SXT/CED/AMX | U | 1 | 0.39 | 1/102 | 0.98 |
| 21402 | FLR/ATM/TCY/CED/AMX | V | 1 | 0.39 | 1/102 | 0.98 |
| 21617 | FLR/ATM/MEM/CED/AMX | W | 1 | 0.39 | 1/102 | 0.98 |
| 21622, 21406, 20504 | FLR/MEM/SXT/TCY/CED/AMX | X | 3 | 0.46 | 3/102 | 2.94 |
| 201006 | MEM/CIP/SXT/AMK/TCY/CED/AMX | Y | 1 | 0.54 | 1/102 | 0.98 |
| 21502 | FLR/MEM/CIP/SXT/TCY/CED/AMX | Z | 1 | 0.54 | 1/102 | 0.98 |

**Point 14.** The detection of virulence and antimicrobial resistance genes in the recovered isolates should be performed. Afterwards, the correlation between phenotypic and genotypic multidrug resistance should be performed.

Besides, the correlation coefficient (r) should be determined among the identified resistance genes and the tested antimicrobial agents.

**Response 14:** As in ‘Point 19-20’, We have corrected as Reviewer’s suggestion.

**Point 15.** Where are the Statistical analyses? Add more details about the used software.

**Response 15:** We are grateful for the suggestion. To be more clear and by the Reviewer concerns, we have added:

(Page 11, Line 293-300):

**Statistical analyses**

“The experimental data was analyzed using SPSS 20 software. Statistical analysis was performed using the Pearson correlation method to test the correlation coefficient between drug-resistance genotype and phenotype. The statistical significance level is set at a two-tailed α-value of 0.05, therefore, p-value less than 0.05 is considered statistically significant Multidrug-resistant strains are resistant to three or more antibiotics [45, 59]. The calculation formula of Multi antibiotic resistant MAR index was calculated by using the follow formula: MAR = Number of antibiotics to which an isolate showed resistance / Total number of antibiotics used in the experiment [60].”

**Results:**

**Point 16.** Please add a starting paragraph to the results section to briefly introduce the topic, your goals and hypothesis and a short summary of what you did in this work.

**Response 16:** As ‘Point 2’, We have revised a paragraph corresponding to line 32-57 of page 2 to the Abstracts (Page 2, Line 32-57)

**Point 17.** Add this subtitle: Phenotypic characteristics of the recovered isolates:

**Point 18.** Illustrate in detail the phenotypic characteristics of the recovered A. hydrophila and A. veronii isolates.

**Response 17-18:** We have added a paragraph corresponding to line 444-451 of page 16-17 as follows:

(Page 16-17, Line 444-451):

**Drug-resistant phenotypes of the recovered isolates**

“The relationship between drug resistance and species of isolates was analyzed by using the WHONET software. The drug-resistant phenotype of *A. sobria* was type X according to the spectrum; The drug-resistant phenotype of *A. caviae* was type A; *A. salmonicida* contains four drug-resistant phenotypes type A, B, G, and I; *A. hydrophila* contained four phenotypes, type A, B, I and M; *A. veronii* contains all spectrum type A-Z. It can be seen that there is a large difference in the number of samples. Except for type A and B, the frequency of other drug-resistant phenotypes has no obvious correlation with strains.”

**Point 19.** Antimicrobial susceptibility testing

Illustrate in a new table the occurrence of MDR (Multidrug resistance) among the recovered isolates as the following (illustrate the names of the antimicrobial classes and different antibiotics):

No. of strains % Type of resistance R, MDR, and XDR Phenotypic multidrug resistance (Antimicrobial classes and different antibiotics). The antibiotic-resistance genes

**Point 20.** The correlation (Correlation coefficient) between phenotypic and genotypic multidrug resistance should be performed.

**Response 19-20:** We appreciate the Reviewer’s positive evaluation of our work and agree with the comments regarding the limitations of our study. We have created a new “Table 4”, however, as the data related to drug resistance genes has already been recorded in “Table 5”, genotype information has not been included in this modification for the time being, instead, we have added MAR index according to your suggestion:

(Page 14, Line 381-386): “The resistance spectrum analysis of 102 *Aeromonas* isolates showed that they are resistant to at least one and up to seven antibiotics (Table 4). The range of multi antibiotic resistant (MAR) index was from 0.08 to 0.54. The two isolates with the highest MAR index are strain 201006 and 21502 which exhibited resistant to seven antibiotics. The MAR index of 18 strains was 0.08, indicating these strains were resistant to at least one type of antibiotic. 44 out of 102 *Aeromonas* isolates were resistant to at least three antibiotics, and the MAR index was higher than 0.23.”

**Point 21.** Increase the resolution of figures (must be 600 dpi).

**Response 21:** We have corrected as Reviewer’s suggestion. All figures (1-7) have been recreated in HD.

**Discussion:**

**Point 22.** Please illustrate different mechanisms of antimicrobial resistance in Aeromonas spp.

**Response 22:** We sincerely appreciate the valuable comments. We have revised the Introduction corresponding to line 64-75 of page 3 as follows:

(Page 3, Line 64-75): “*Aeromonas* spp. possess various virulence factors such as degradative enzymes, surface polysaccharides (capsule, lipopolysaccharide, and glucan), S-layers, iron-binding systems, exotoxins, secretion systems, fimbriae, other nonfilamentous adhesins, and flagella [3, 4]. Various virulence factors of *Aeromonas* spp. cause immunocompromised organisms through resistance to host defense mechanisms, leading to diseases such as septicemia, ulcers, and visceral erosion and directly damaging host cells, resulting in *Aeromonas* infection and cell death [5, 6]. In addition, antibacterial and antioxidant molecules in the fish body provide self-defense against invading *Aeromonas* spp. and virulence factors, but the end product of this is reactive oxygen species (ROS), which can cause host cell damage and stress [7]. Aquatic animals such as fish, shrimp, and amphibians are particularly susceptible to *Aeromonas* infection due to *Aeromonas* spp. being the most widespread species in the aquatic environment, which can result in high mortality [8, 9].”

**Conclusion:**

**Point 23.** Should be rephrased to be sounded. A real conclusion should focus on the question or claim you articulated in your study, which resolution has been the main objective of your paper?

**Response 23:** We have revised the Materials and Methods corresponding to line 701-712 of page 26 as follows:

(Page 26, Line 701-712): “In conclusion, further investigation of resistance factors at the species level, including strains, habitats, and temperature, is required to reveal the drug resistance and infection mechanisms of *Aeromonas* [71]. A deeper mechanism study requires an accurate and efficient classification method for multidrug-resistant *Aeromonas* species. Therefore, we wanted to confirm that the MLST method (multiple concatenated gene loci) was more helpful for accurate classification than the previously used single gene (*16S rRNA*, *gyrB* gene) method. Therefore, multidrug-resistant *Aeromonas* obtained from fish living in Hebei Province, China, were classified using two methods. The MLST method is suitable to contribute to the classification of multidrug-resistant *Aeromonas* species and provides theoretical information for multidrug-resistant *Aeromonas* infection prevention and antibiotic selection in the aquaculture industry in Hebei Province. The findings of this study will serve as a foundation for genetic research on the clear infection mechanism and antibiotic resistance mechanism of *Aeromonas* according to environmental conditions.”
