# Supplementary material for: Multilocus sequence typing and antibiotic resistance of Aeromonas isolated from freshwater fish in Hebei Province
Source: PLoS One. 2024 Mar 27;19(3):e0298745. doi: 10.1371/journal.pone.0298745 (PMC10971578; doi:10.1371/journal.pone.0298745)
Supplement: Attachment — Submitted filename: Author to respond reviewer 3 - R2.docx [file pone.0298745.s008.docx]

**Response to Reviewer 3 Comments – R2**

This study proposed that the identification selective power of Aeromonas spp. might be increased by employing the Multilocus Sequence Typing technique. However, this is an interesting work. But it has some challenges that need to be addressed before it is ready for publication:

**Results:**

**Point 1.** Line 402: remove the reference (61), Page 15.

**Point 2.** Line 413: remove the reference (32), Page 16.

**Point 3.** Line 572: remove the reference (62), Page 21.

**Response 1-3:** We thank the Reviewer for pointing this out. We have corrected as Reviewer’s suggestions.

(Page 14, Line 367): [61] Huys G, Cnockaert M, Swings J. Aeromonas culicicola Pidiyar et al. 2002 is a later subjective synonym of Aeromonas veronii Hickman-Brenner et al. 1987 [J]. Systematic and applied microbiology, 2005, 28: 604-609.

(Page 14, Line 372): [32] The Clinical and Laboratory Standards Institute Subcommittee on Antimicrobial Susceptibility Testing: Background, Organization, Functions, and Processes

(Page 19, Line 525): [62] Coffey T J, Pullinger G D, Urwin R, et al. First Insights into the Evolution of Streptococcus uberis: a Multilocus Sequence Typing Scheme That Enables Investigation of Its Population Biology [J]. Applied & Environmental Microbiology, 2006, 72(2): 1420-1428.
