# Supplementary material for: Multilocus sequence typing and antibiotic resistance of Aeromonas isolated from freshwater fish in Hebei Province
Source: PLoS One. 2024 Mar 27;19(3):e0298745. doi: 10.1371/journal.pone.0298745 (PMC10971578; doi:10.1371/journal.pone.0298745)
Supplement: Attachment — Submitted filename: Reviewer comments.docx [file pone.0298745.s005.docx]

**Reviewer’s comments**

This study proposed that the identification selective power of Aeromonas spp. might be increased by employing the Multilocus Sequence Typing technique. However, this is an interesting work. But it has some challenges that need to be addressed before it is ready for publication:

**Abstract**

- Line 40-41: A total of 130 Aeromonas spp. isolates were isolated from freshwater fish farms in Hengshui, Handan, and Shijiazhuang and sequenced for species identification. Did you sequence all these 130 isolates? Need to be corrected.
- Line 44: using the MLS, it should be Multilocus Sequence Typing (MLST).
- line 46: what is the abbreviation Sts referee to? (sequence types).
- line 70: need reference.
- line 73: The occurrence of various diseases caused by Aeromonas spp. has (have) prompted.
- line 108: The Clinical and Laboratory Standards Institute (CLSI) [21]. Should be the updated reference for CLSI.

**Materials and methods**

- You should clarify the fish sampling method, and type of fish collected (healthy, moribund, freshly dead??). under title of (2.1. fish samples collection).
- Line 118: Samples were collected from the body surface and affected areas. Should be declared what is the clinical picture of the affected areas?? Or the affected areas are the localities suffered from outbreaks? This is confusing!!
- Line 120: the samples were (aseptically) cultured (on) not in.
- Line 121: RS media should include the manufacturer company.
- Line 123: The boiling method. You should cite a reference for this technique.
- Line 124: Single-colony isolates!! What does it mean? Need clarification.
- Line 124: Brain heart infusion broth (BHIB) should include the manufacturer company.
- Line 125: add (at) before 30°C.
- Line 129: The supernatant was collected for genomic DNA!! The supernatant directly collected after boiling or centrifugated prior collection?
- Line 134: The *16S r*RNA primer. Is this primer universal for Aeromonas spp. Or universal Bacterial primer?? Should be clarified.
- Also, these primers are designed or used according to references that must be cited.
- Line 162: Kirby-Bauer disk diffusion susceptibility test, need a reference for this technique.
- Line 165: Cotrimoxazole, should be co-trimoxazole.
- Line 169: The 102 *Aeromonas* isolates!! What was happened to the rest of the remaining isolates (130)? And why you select these isolates??
- Line 169: were inoculated on BHIB agar, BHI (Broth or agar)??
- Line 171: Coated in MHA medium, coated on MHA medium, should fully written (Muller Hinton Agar) and include the manufacturer company.
- What is the dose of bacterial suspension inoculated on agar?
- Why you incubate at 28°C not at the same isolation temperature?
- Line 172: CLSI, should be cited here.
- Line 196: Multilocus Sequence Typing (MLST) was conducted on 47 *Aeromonas* strains out of 102 total isolates??? And why you select these isolates??, need to be clarified.
- Line 198: (Table 7), must not be cited in the material and method section.
- The ethical approval statement should be written.

**Results:**

- Line 263: 104 isolates or as mentioned before 102?. Need to be revised.
- Line 271: didn’t you identified *A. sobria* while using the *16S rRNA* primers????. How it did not identified with the *16s r*RNA universal bacterial primer?. Need explanation.
- Line 293-300: must be in discussion section not in results.
- Line 307: not in results section.
- Line 328: what is NJ method means?
- Lines 328-330: In general, ML method perform better than NJ method when utilized by appropriate nucleotide substitution models [41]. However, the actual construction shows that the difference between the tree construction based on the two different algorithms is relatively tiny for single gene. (this in discussion section not results!).
- Line 339: We don’t cite references in the result section!!!!!
- Lines 338-334: Must be in discussion not in the result section.
- Line 348: the CLSI-2020, must be cited in the text and in the references list.
- Line 349: The rate of drug resistance to amoxicillin was the highest (92.2%)!!!!! Not 100%??
  need to be revised as Aeromonas bacteria carry resistance gene for all beta lactams.
  Line 367: All 102 *Aeromonas* isolates were resistant to amoxicillin, while in line 349: The rate of drug resistance to amoxicillin was the highest (92.2%)!!!!!
  this is confusing, need to be explained.
- Lines 539-543: This paragraph must be in discussion not in the result section.
- Line 866: Table 3. Antibiotic and drug resistance profile, Amoxicillin 92.2% R?????
