# Supplementary material for: Multilocus sequence typing and antibiotic resistance of Aeromonas isolated from freshwater fish in Hebei Province
Source: PLoS One. 2024 Mar 27;19(3):e0298745. doi: 10.1371/journal.pone.0298745 (PMC10971578; doi:10.1371/journal.pone.0298745)
Supplement: Attachment — Submitted filename: Reviewer comments - R2.docx [file pone.0298745.s007.docx]

**Reviewer’s comments (R2)**

Dear authors, in the Results section, we focus exclusively on presenting our own findings without including results from other studies or any references, thus address the following comments;

**Results:**

Line 402: remove the reference (61), Page 15.

Line 413: remove the reference (32), Page 16.

Line 572: remove the reference (62), Page 21.
